# Bacterial Genotoxin-Coated Nanoparticles for Radiotherapy Sensitization in Prostate Cancer

**DOI:** 10.3390/biomedicines9020151

**Published:** 2021-02-04

**Authors:** Yu-An Chen, Yi-Ru Lai, Hui-Yu Wu, Yen-Ju Lo, Yu-Fang Chang, Chiu-Lien Hung, Chun-Jung Lin, U-Ging Lo, Ho Lin, Jer-Tsong Hsieh, Cheng-Hsun Chiu, Yu-Hsin Lin, Chih-Ho Lai

**Affiliations:** 1 Department of Microbiology and Immunology, Graduate Institute of Biomedical Sciences, Chang Gung University, Taoyuan 33302, Taiwan; yu-an.chen@utsouthwestern.edu (Y.-A.C.); d0801202@cgu.edu.tw (Y.-R.L.); winney0614@gmail.com (H.-Y.W.); andy56320218@gmail.com (Y.-J.L.); yufang1825128@gmail.com (Y.-F.C.); 2 Department of Life Sciences, National Chung Hsing University, Taichung 40227, Taiwan; hlin@dragon.nchu.edu.tw; 3 Department of Urology, University of Texas Southwestern Medical Center, Dallas, TX 75390, USA; sunshinegrass@yahoo.com.tw (C.-J.L.); U-Ging.Lo@utsouthwestern.edu (U.-G.L.); jt.hsieh@utsouthwestern.edu (J.-T.H.); 4 Targeted Drug and Delivery Technology Division, Biomedical Technology and Device Research Laboratories, Industrial Technology Research Institute, Hsinchu 30011, Taiwan; joyhung@itri.org.tw; 5 Department of Medical Research, School of Medicine, China Medical University and Hospital, Taichung 40447, Taiwan; 6 Molecular Infectious Disease Research Center, Department of Pediatrics, Chang Gung Memorial Hospital, Linkou 33305, Taiwan; 7 Center for Advanced Pharmaceutics and Drug Delivery Research, Department and Institute of Pharmacology, Institute of Biopharmaceutical Sciences, Faculty of Pharmacy, National Yang Ming Chiao Tung University, Taipei 11221, Taiwan; 8 Department of Nursing, Asia University, Taichung 41354, Taiwan

**Keywords:** genotoxin, nanoparticles, prostate cancer, radioresistance

## Abstract

Prostate cancer (PCa) is one of the most commonly diagnosed cancers in men and usually becomes refractory because of recurrence and metastasis. CD44, a transmembrane glycoprotein, serves as a receptor for hyaluronic acid (HA). It has been found to be abundantly expressed in cancer stem cells (CSCs) that often exhibit a radioresistant phenotype. Cytolethal distending toxin (CDT), produced by *Campylobacter jejuni*, is a tripartite genotoxin composed of CdtA, CdtB, and CdtC subunits. Among the three, CdtB acts as a type I deoxyribonuclease (DNase I), which creates DNA double-strand breaks (DSBs). Nanoparticles loaded with antitumor drugs and specific ligands that recognize cancerous cell receptors are promising methods to overcome the therapeutic challenges. In this study, HA-decorated nanoparticle-encapsulated CdtB (HA-CdtB-NPs) were prepared and their targeted therapeutic activity in radioresistant PCa cells was evaluated. Our results showed that HA-CdtB-NPs sensitized radioresistant PCa cells by enhancing DSB and causing G2/M cell-cycle arrest, without affecting the normal prostate epithelial cells. HA-CdtB-NPs possess maximum target specificity and delivery efficiency of CdtB into the nucleus and enhance the effect of radiation in radioresistant PCa cells. These findings demonstrate that HA-CdtB-NPs exert target specificity accompanied with radiomimetic activity and can be developed as an effective strategy against radioresistant PCa.

## 1. Introduction

Prostate cancer (PCa) is a threatening malignancy that has demonstrated an escalating trend in both incidence and mortality rate worldwide in recent years [1]. Patients may develop several non-specific symptoms such as urinary retention, urinary hesitancy, nocturia, and hematuria [2]. Because of the high heterogeneity of PCa, the relationship between certain symptoms and cancer development is rather weak and undefined [3]. However, attributed to the advancement in diagnostic tests, more people opt for clinical management during the early phases. Treatment strategies for PCa include surgery, chemotherapy, hormone therapy, and radiation therapy [4]. Among these, radiation therapy is considered as an effective remedy for localized cancers, which improves prognosis when combined with other appropriate treatment modalities [5,6]. Therefore, the unpredictable emergence of radioresistance may result in a setback for successful treatments. Furthermore, the development of radioresistance often leads to tumor metastasis or relapse [7]. To overcome this problem, a radiation sensitizer is likely to be the solution.

Cytolethal distending toxin (CDT), a genotoxin produced by *Campylobacter jejuni* (*C. jejuni*), causes DNA double-strand breaks (DSBs) [8]. CDT comprises three subunits, CdtA, CdtB, and CdtC, which are encoded by the consecutive genes *cdtA*, *cdtB*, and *cdtC*, respectively [9]. The working subunit, CdtB, acts as a DNase I, which is responsible for creating DSB, cell cycle arrest at the G2/M phase [10], thereby inducing cell distension and ultimately cell death [11]. However, CdtB cannot traverse the cell membrane alone and, therefore, its entry into the cytoplasm requires the binding of CdtA and CdtC to the cell membrane. With the help of the nuclear localization signal (NLS), CdtB is subsequently translocated into the nucleus [12]. Given the similarity of CDT with radiation, wherein both elicit cell death by causing DSBs, it is highly possible for CDT to be an ideal radiation sensitizer.

Although CDT appears to be a promising cure for radioresistant PCa, the specific delivery of CDT to PCa cells remains a critical issue. This challenging task can be overcome by utilizing the CD44-overexpressing nature of PCa cells [13]. CD44 is a transmembrane glycoprotein which mediates extracellular adhesion and signal transduction [14]. In addition, CD44 has been involved in tumor progression and metastasis, drug resistance, and radiation resistance of cancer cells [15,16]. Moreover, nanoparticles have gained growing attention in pharmaceutical research [17]. Several studies have demonstrated that using hyaluronic acid (HA)-decorated nanoparticles to encapsulate various therapeutic drugs could effectively target CD44-overexpressing cells and subsequently deliver the encapsulated agents into those cells [18,19,20]. HA, also known as hyaluronan, is a naturally occurring non-sulfated glycosaminoglycan, with characteristics such as biocompatibility, non-immunogenicity, and biodegradability, making it an ideal material for targeting CD44^+^ cancer cells [20].

In this study, HA-decorated nanoparticle-encapsulated CdtB (HA-CdtB-NPs) were developed and extensively studied for their characteristics, including the particle size, loading efficiency, and zeta potential. PCa cells with DOC-2/DAB2 interactive protein (DAB2IP) knockdown were chosen to imitate the cancer stem cells (CSC) with radioresistant phenotype [21]. After treating PC3 DAB2IP-knockdown (PC3-KD) cells with HA-CdtB-NPs, the drug efficacy, cytotoxicity, and delivery efficiency were assessed.

## 2. Materials and Methods

### 2.1. Antibodies and Reagents

Antibodies against cleaved-PARP were purchased from Proteintech (Chicago, IL, USA). Antibodies specific to phospho-H2AX and phospho-CHK2 were purchased from Cell Signaling (Danvers, MA, USA). Antibodies against β-actin, proliferating cell nuclear antigen (PCNA), and rabbit horseradish peroxidase (HRP)-conjugated antibody were purchased from Santa Cruz Biotechnology (Santa Cruz, CA, USA). Antibody against CD44 was purchased from Novus Biologicals (Centennial, CO, USA). Other reagents and chemicals were purchased from Sigma-Aldrich (St. Louis, MO, USA).

### 2.2. Purification of CDT Subunits

Recombinant His-tagged CdtA, CdtB, and CdtC subunits were constructed as described in our previous study [22]. A single colony of *Escherichia coli* BL21-DE3 containing *cdtA*, *cdtB*, *cdtC* expression plasmids, respectively, was picked and cultured in LB broth with ampicillin (100 μg/mL) at 37 °C. *E. coli* harboring *cdtA*, *cdtB*, *cdtC* recombinant plasmids were then induced by isopropyl β-D-1-thiogalactopyranoside (IPTG) at 37 °C for 5 h for those containing *cdtA* and *cdtB*, and at 16 °C for 16 h for those containing *cdtC*, respectively. Recombinant proteins were purified by metal affinity chromatography (Clontech, Mountain View, CA, USA) and analyzed by SDS-PAGE and Western blot assay.

### 2.3. Preparation of HA-Decorated Nanoparticle-Encapsulated CdtB

Recombinant CdtB was purified according to the protocol described above. To prepare HA-decorated nanoparticle-encapsulated CdtB, a simple ionic gelation with magnetism was performed at room temperature. The determination of the optimal preparation conditions was performed by examining different proportions of HA/arginine-chitosan (HA/Arg-CS). In brief, aqueous HA (4.00 mg/mL, 0.50 mL) was added into aqueous Arg-CS with various concentrations (1.00, 2.00, 3.00, or 4.00 mg/mL, 0.50 mL), and then the solutions were gently shaken for 30 min at 37 °C. After collecting the prepared HA/Arg-CS NPs produced by centrifugation, a Zetasizer instrument was used to measure the particle size, polydispersity index, and zeta potentials. Meanwhile, the NP suspension was placed onto a mesh copper grid and positively stained with osmium tetroxide to observe their morphology under transmission electron microscopy.

Next, different concentrations of CdtB were encapsulated in nanoparticles to determine the optimal condition for preparing CdtB-loaded HA/Arg-CS NPs. CdtB dissolved in deionized water (3.00, 2.00, 1.00 mg/mL; 0.25 mL) was mixed with HA solution (8.00 mg/mL; 0.25 mL) to form CdtB/HA aqueous mixed solutions (1.50:4.00, 1.00:4.00, and 0.50:4.00 mg/mL, 0.50 mL). The CdtB/HA solutions (0.50 mL) were added to the optimal concentration of Arg-CS in deionized water (0.50 mL) and stirred at 37 °C for 30 min. After centrifugation, the amount of free CdtB in the supernatant was detected by a protein concentration assay (Bio-Rad, Hercules, CA, USA) and protein-loading efficiency of the NPs was calculated from the following equation:

$$\mathrm{Loading}\;\mathrm{efficiency}\;=\;\frac{\mathrm{Total}\;\mathrm{CdtB}\;\mathrm{protein}\;-\;\mathrm{free}\;\mathrm{CdtB}\;\mathrm{protein}}{\mathrm{Total}\;\mathrm{CdtB}\;\mathrm{protein}}\times \;100\%$$

### 2.4. Cell Culture

Human prostate epithelial cell line (PZ-HPV-7) was derived from the peripheral zone of a benign prostate and cultured as described previously [23]. PC3-KD cells, the prostate cancer cells with knockdown of endogenous DOC-2/DAB2 interactive protein (DAB2IP), were described previously [24]. PC3-KD (knockdown DAB2IP) cells were cultured in Roswell Park Memorial Institute (RPMI) 1640 medium (Hyclone, Logan, UT, USA) with 5% fetal bovine serum (FBS) (Hyclone) containing puromycin (0.4 μg/mL), 1× penicillin/streptomycin, and G418 (800 μg/mL) in the environment of 37 °C and 5% CO_2_.

### 2.5. Analysis of Cell Viability

PC3-KD cells were seeded in 96-well plates and treated with 0, 10, 50, 100, 200, 500, and 1000 nM of HA/Arg-CS (HA-NPs), CDT holotoxin and HA-CdtB-NPs, respectively. After incubation for 48 h, cells were treated with 100 μL of 0.5 mg/mL 3-(4,5-dimethylthiazol-2-yl)-2,5-diphenyltetrazolium bromide (MTT) solution in 37 °C for 2 h. Cell viability was analyzed by measuring the ability of viable cells reducing MTT (Sigma-Aldrich) to formazan [25].

### 2.6. HA-CD44 Localization and DNA Double-Strand Break (DSB) by Immunofluorescence

PC3-KD cells were seeded onto cover glasses and incubated for 24 h. For DSB analysis, cells were first treated with 2 Gy ionizing radiation (IR), then exposed to 200 nM of HA-NPs, CDT holotoxin, and HA-CdtB-NPs, respectively. Cells were subsequently fixed with 1% paraformaldehyde for 1 h, permeabilized by 0.1% triton X-100 for 15 min. For the HA-CD44 colocalization assay, cells were then treated with primary antibody against CdtB. For DSB analysis, cells were treated with anti-pH2AX and anti-53BP1 at the dilution of 1:200, followed by treatment with secondary antibody at the dilution of 1:500 and 4′,6-diamidino-2-phenylindole DAPI (0.2 μg/mL). The stained cells were observed using a Laser Scanning Confocal Microscope (LSM780, ZEISS, Jena, Germany).

### 2.7. Analysis of CdtB in the Nuclear Extraction

PC3-KD cells were treated with 100 nM HA-NPs, CDT holotoxin, and HA-CdtB-NPs, followed by incubation for 0, 0.5, 1, 3, and 6 h. The nuclear proteins were isolated using a nuclear extraction kit (Pierce, Rockford, IL, USA), as described previously [22]. CdtB level in the nuclear fraction was then analyzed by Western blot assay.

### 2.8. Western Blot Analysis

PC3-KD cells were seeded onto a 60-mm dish and incubated at 37 °C for 16 h, then treated with medium (mock), IR, HA-NPs accompanied by IR, CDT holotoxin accompanied by IR, and HA-CdtB-NPs accompanied by IR, respectively, for 24 and 48 h. Cell lysates were centrifuged at 12,000 rpm for 20 min at 4 °C. After quantification, samples were resolved by 12% SDS-PAGE and transferred onto polyvinylidene difluoride membranes (Millipore, MA, USA). The polyvinylidene fluoride (PVDF) membrane was blocked and probed with primary antibodies against (ADP-Ribose) P polymerase (PARP), pH2AX, pATM, pCHK2, and β-actin in the condition of gentle shaking. After overnight incubation at 4 °C, the membrane was then probed with horseradish peroxidase-conjugated secondary antibody (Millipore) at room temperature for 1 h. The proteins of interest were detected using ECL Western Blotting Detection Reagents (GE Healthcare, Piscataway, NJ, USA) and were visualized using an Azure c400 system and AzureSpot Analysis Software (Azure Biosystems, Dublin, CA, USA) by following the manufacturer’s instructions.

### 2.9. Cell Cycle Analysis by Flow Cytometry

PC3-KD cells were incubated with ICRF-193 (2 μg/mL), HA-CdtB-NPs (50 nM, 100 nM, and 200 nM), and HA-NPs alone for 48 h. The cells were washed with PBS and centrifuged for 1000 rpm at room temperature for 5 min. Subsequently, the prepared cells were resuspended in hypotonic buffer (0.2 mg/mL RNase A, 20μg/mL propidium iodide, and 0.1% Triton X-100). Analysis of cell cycle was performed by flow cytometry (Becton Dickinson, San Diego, CA, USA).

### 2.10. Comet Assay

Experimental protocols followed the instructions of the Trevigen CometAssay^®^ Kit (Trevigen, Gaithersburg, MD, USA). PC3-KD cells were seeded in a 60-mm dish and respectively treated with medium (mock), HA-NPs, CDT holotoxin, and HA-CdtB-NPs, with three of the latter accompanied with IR. After incubation for 24 h, PC3-KD cells (1 × 10^5^/mL) were first mixed with LMAgarose with the proportion of 1:10 and placed onto CometSlide^TM^ with complete coverage of the sample area; then, the slide was incubated at 4 °C in the dark for 30 min. Cells were then immersed with 4 °C lysis solution at 4 °C for 30–60 min, followed by alkaline unwinding solution at 4 °C in the dark for 1 h. CometSlide^TM^ were placed in alkaline electrophoresis solution and the power supply set to 21 volts; then, the voltage was applied for 30 min and cells were washed by distillation-distillation H_2_O (ddH_2_O) and 70% alcohol. After CometSlide^TM^ were dried and stained by CYBR^®^ Gold, cells were observed using Leica CTR 4000 (Leica, Wetzlar, Germany).

### 2.11. Statistical Analysis

The analysis among multiple groups was carried out by means of one-way analysis of variance. The post-hoc test was used to measure the statistical significance of differences between two groups. Statistical analyses and chart drafting were carried out using the GraphPad Prism 6 software (GraphPad Software Inc., San Diego, CA, USA).

## 3. Results

### 3.1. Preparation and Characterization of CDT and HA-CdtB-NPs

**Figure 1:**
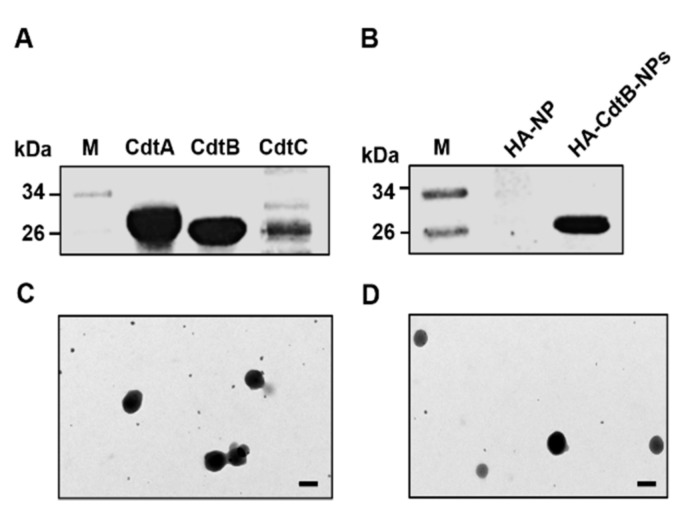
Characterization of HA-CdtB-NPs. SDS-PAGE analysis of (**A**) each recombinant CDT subunit and (**B**) HA-CdtB-NPs. Transmission electron micrographs of (**C**) HA-NPs and (**D**) HA-CdtB-NPs. Scale, 200 nm.

Purified recombinant CDT subunits and synthesized HA-CdtB-NPs were analyzed by SDS-PAGE (Figure 1A,B). The NPs were constructed with negatively charged HA and positively charged arginine-chitosan (Arg-CS). As shown in Table 1, HA:Arg-CS used in distinct concentrations (2.0:0.5, 2.0:1.0, and 2.0:2.0 by mg/mL) formed complexes on the nanometer scale, with the exception of the concentration 2.0:1.5 mg/mL. The diameters of the prepared NPs were in the range of 220–300 nm (Table 1 and Figure 1C), and the negatively or positively charged values were dependent on the relative concentrations of HA and Arg-CS. When the amount of the positively charged Arg-CS sufficiently exceeded that of the negatively charged HA (HA:Arg-CS = 2.0:2.0 mg/mL), the NPs had Arg-CS exposed on their surfaces and, thus, had a positive surface charge. In contrast, when the amount of the negatively charged HA significantly exceeded that of the positively charged Arg-CS (HA:Arg-CS = 0.5:2.0 or 1.0:2.0 by mg/mL), some of the excessive HA molecules were entangled onto the surfaces of the obtained NPs. Thus, the resulting NPs displayed a negative surface charge. The HA/Arg-CS concentration of 2.0:0.5 mg/mL produced the mean particle size of 225.64 ± 14.65 nm with a maximum negative zeta potential of −38.79 ± 4.51 mV (*n* = 5). We then characterized CdtB-loaded nanoparticles by Fourier transform infrared spectroscopy (FTIR) analysis. The results showed that Arg-CS peaks at 1145 and 770 cm^−1^, corresponding to asymmetric C-C-N bond bending and COO^−^ scissoring modes of Arg, respectively, and a characteristic band at 1558 cm^−1^ representing bending associated with protonated amino groups (–NH_3_^+^) of CS [26]. The HA spectrum exhibited C=O asymmetric stretching at 1610 cm^−1^ and C–O symmetric stretching at 1406 cm^−1^, corresponding to carboxyl groups [27,28]. The Arg-CS/HA nanoparticle spectrum shifted characteristic protonated amino groups in CS at 1558 cm^−1^ and carboxyl ion peaks in HA at 1610 cm^−1^ to 1562 and 1615 cm^−1^, respectively (Figure S1). Ionized Arg-CS and HA therefore formed polyelectrolyte complexes via electrostatic interactions, and the NPs prepared with this specific composition were used for the following studies.

In order to prepare the CdtB-encapsulated NPs, various concentrations of CdtB were mixed with HA/Arg-CS encapsulated mixture solution. The HA/Arg-CS nanoparticle-encapsulated CdtB is attributed to an electrostatic interaction between the negative charge on CdtB (zeta potential of −10.86 ± 3.95 mV) and the positive charge on chitosan (zeta potential of +35.67 ± 5.79 mV). As shown in Table 2 and Table S1, the CdtB:HA:Arg-CS in distinct concentrations showed a mean size range of 250–300 nm, with different negative potential values. When the concentration of CdtB:HA:Arg-CS is 0.75:2.00:0.50 mg/mL, the loading efficiency of CdtB in NP is 48.97 ± 5.12%, its particle size is 273.78 ± 17.37 nm, zeta potential value is −39.89 ± 9.65 mV. In addition, the polydispersity index of NPs measured using dynamic light scattering (DLS) revealed a narrow distribution (polydispersity index: 0.27 ± 0.06) and the morphology of the prepared NPs along with CdtB-encapsulated NPs remained spherical and smooth-shaped on transmission electron microscopy observation (Figure 1D). Therefore, the HA-decorated NPs encapsulating CdtB with a negative surface charge at a CdtB:HA:Arg-CS concentration of 0.75:2.00:0.50 mg/mL were used for the remaining studies.

### 3.2. HA-CdtB-NPs Induce Radioresistant PCa Cell Death

**Figure 2:**
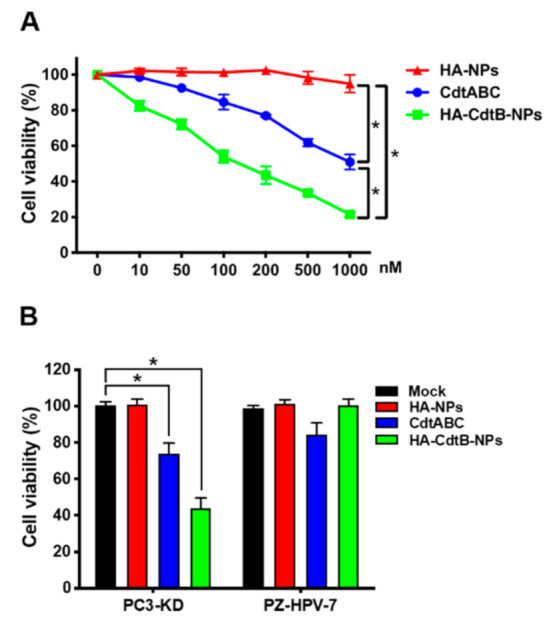
HA-CdtB-NPs inhibit PCa cell proliferation. (**A**) PC3-KD cells were treated with HA-NPs, CDT holotoxin, or HA-CdtB-NPs at the indicated concentrations (0–1000 nM) for 48 h. (**B**) PZ-HPV-7 and PC3-KD cells were treated with HA-NPs, CDT holotoxin, or HA-CdtB-NPs at the concentration of 100 nM for 48 h. Cell viability was assessed using MTT assay. Statistical significance was evaluated using one-way ANOVA with post hoc test (* *p* < 0.05).

Loss of DOC-2/DAB2 interactive protein (DAB2IP) expression in PCa cells leads to the development of radioresistance [24]. We therefore utilized PC3-KD (DAB2IP-knockdown) cells as the research platform to investigate the therapeutic effects of HA-CdtB-NPs on radioresistant PCa cells. We first analyzed the CD44 expression in PCa cells and normal prostate epithelial cells. As shown in Figure S2, PCa cells (PC3-KD) express higher levels of CD44 than normal cells (PZ-HPV-7, a prostate epithelial cell line). For evaluating the ability of HA-CdtB-NPs to induce PCa cell death, we performed an MTT assay to analyze the effect of HA-NPs, CDT holotoxin, and HA-CdtB-NPs on the cell viability of the PC3-KD cells and PZ-HPV-7 cells. To determine the ideal conditions for the following experiments, we treated PC3-KD cells with various concentrations of the HA-CdtB-NPs and found that the viable cell numbers dropped to approximately half at a concentration of 100 nM (Figure 2A). Meanwhile, CDT holotoxin treatment also exhibited a reduction effect in a concentration-dependent manner; however, it was less effective than HA-CdtB-NPs. Furthermore, it was denoted that HA-NPs barely affected both PC3-KD and PZ-HPV-7 cells (Figure 2B). Most importantly, PZ-HPV-7 cells did not show any evident response to HA-CdtB-NP treatment, which indicated that HA-CdtB-NPs are able to specifically target PCa cells.

### 3.3. Delivery of HA-CdtB-NPs into PCa Cells

**Figure 3:**
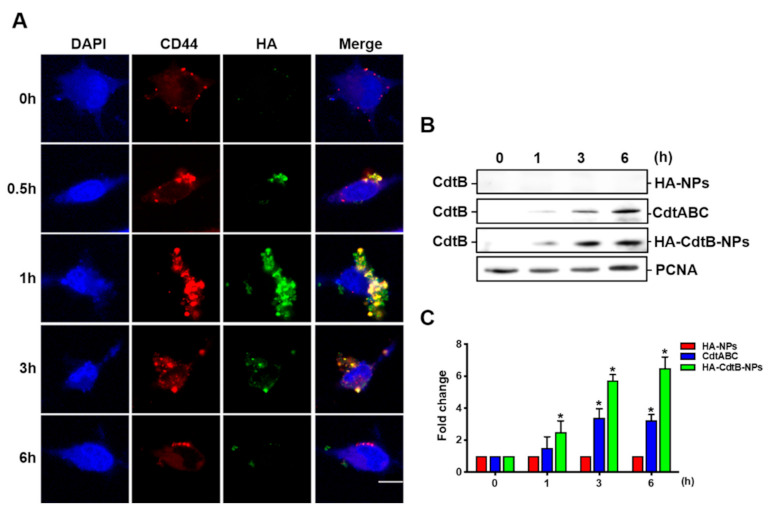
Delivery of HA-CdtB-NPs into PCa cells. (**A**) PC3-KD cells were treated with HA-CdtB-NPs-GFP for 0, 0.5, 1, 3, and 6 h. The colocalization of CD44 and HA on the cell membrane was observed. Scale, 10 μm. (**B**) PC3-KD cells were exposed to 100 nM HA-NPs, CDT holotoxin, and HA-CdtB-NPs, then incubated for the indicated times. The nuclear fraction from cell lysates was prepared and CdtB in the nucleus was analyzed by Western blotting. Proliferating cell nuclear antigen (PCNA) was used as a loading control for nuclear protein. (**C**) The expression of CdtB in the nucleus was quantified for each time point. Statistical significance was evaluated using one-way ANOVA with post hoc test (* *p* < 0.05).

To assess the delivery efficiency of HA-CdtB-NPs into PCa cells, we treated PC3-KD cells with HA-CdtB-NP for different time periods and observed the colocalization of CD44 and HA. As shown in Figure 3A, CD44-HA colocalization could be noted at 0.5, 1, and 3 h, with the peak level of colocalization observed at 1 h. In addition, we performed a Western blot analysis for checking the nuclear translocation of CdtB and the expression levels were also quantified (Figure 3B,C). The highest expression of CdtB in the nucleus was observed at 6 h after HA-CdtB-NP treatment. Interestingly, PC3-KD cells treated with HA-CdtB-NPs demonstrated significantly higher expression of CdtB in the nucleus than those treated with CDT holotoxin. The results showed that HA-CdtB-NPs attached to PC3-KD cells primarily at 1 h after the treatment, and at 6 h, most CdtB entered the nucleus. Our results indicated that HA-CdtB-NPs manifested better delivery efficiency of CdtB into the nucleus than CDT holotoxin, which corresponded with the cell viability assay.

### 3.4. HA-CdtB-NPs Enhance IR-Induced DSB

**Figure 4:**
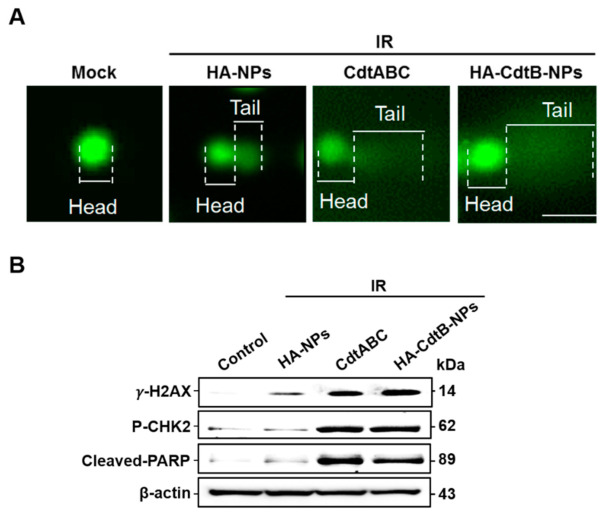
HA-CdtB-NPs enhance IR-induced DSB in PCa cells. PC3-KD cells were mock-treated or exposed to IR (2 Gy) followed by incubation with 100 HA-NPs, CDT holotoxin, and HA-CdtB-NPs. (**A**) Visualization of the comet tail showed IR-induced DSB in PC3-KD cells after 24 h incubation. Scale, 10 μm. (**B**) Western blot analysis for DNA damage-related proteins, γ-H2AX, p-CHK2, and cleaved PARP after 48 h incubation. β-actin was used as a loading control.

**Figure 5:**
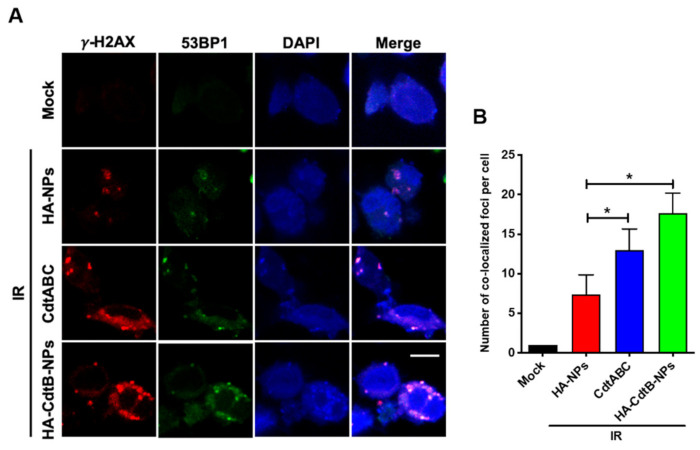
HA-CdtB-NPs sensitize PCa cells to radiation by promoting DSB. PC3-KD cells were mock-treated or exposed to IR (2 Gy) then incubated with 100 nM HA-NPs, CDT holotoxin, and HA-CdtB-NPs for 24 h. (**A**) Fluorescent immunostaining of γ-H2AX (red) and 53BP1 (green) was shown. DAPI (blue) was used as a tracer for cell nucleus. Scale, 10 μm. (**B**) The foci of γ-H2AX and 53BP1 colocalization in the nuclei were counted. Statistical significance was evaluated using one-way ANOVA with post hoc test (* *p* < 0.05).

To examine whether HA-CdtB-NPs regain the sensitivity of PC3-KD cells toward IR, three different procedures that allow the visualization of DSBs were performed, and four conditions were included: mock treatment, HA-NPs/IR cotreatment, CDT holotoxin/IR cotreatment, and HA-CdtB-NPs/IR cotreatment. First, in the comet assay, comet-like structures were observed in the cells for all treatment conditions except for the mock treatment (Figure 4A). Nevertheless, the HA-CdtB-NPs/IR cotreatment displayed a brighter and longer tail than the other treatments. Second, expression levels of γ-H2AX, p-CHK2, and cleaved-PARP were analyzed using Western blot (Figure 4B). HA-NPs/IR cotreatment manifested similarly low expression to the mock treatment. Although the γ-H2AX and p-CHK2 expressions in CDT holotoxin/IR and HA-CdtB-NPs/IR treatments are similar, expression levels of γ-H2AX, p-CHK2, and cleaved-PARP are still the highest in HA-CdtB-NP/IR treatment among all the treating conditions. Finally, as shown by the immunofluorescence staining, the extent of DSB is indicated using the number of foci of γ-H2AX and 53BP1 (Figure 5). Similar to the previous results, the foci were observable in all treatment conditions except for the mock control, and HA-CdtB-NPs/IR cotreatment solely demonstrated maximum foci formation. These results reveal that HA-CdtB-NPs were the most effective and could synergistically enhance IR-induced DSB in radioresistant PCa cells.

### 3.5. HA-CdtB-NPs Induce Cell Cycle Arrest

**Figure 6:**
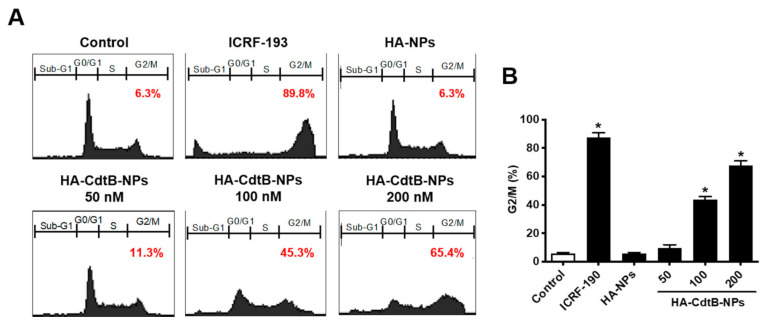
HA-CdtB-NPs induce cell cycle arrest and apoptosis. (**A**) PC3-KD cells were mock-treated, exposed to ICRF-193 or HA-CdtB-NPs (50, 100, and 200 nM), and incubated for 48 h. Cell cycle distribution based on DNA content was analyzed using flow cytometry. (**B**) The percentage of cells at G2/M phase was calculated. Statistical significance was evaluated using one-way ANOVA with post hoc test (* *p* < 0.05).

Since DSB elicits the spontaneous activation of the DNA damage response, which is likely to arrest the cell cycle, we further characterized the effect of HA-CdtB-NPs on the cell cycle distribution. For the control group and HA-NP treatment group, merely 6.29% and 6.25% of cells were arrested at the G2/M phase (Figure 6A,B). ICRF-193 treatment served as a positive control, which arrested 89.82% of cells at the G2/M phase. An apparent increase in the population in the G2/M phase was detected with HA-CdtB-NP treatment; moreover, the increase in HA-CdtB-NP concentration also increased the population at the G2/M phase. These results demonstrate that HA-CdtB-NPs exerted better activity that induced DSBs and cell cycle arrest at the G2/M phase. 

## 4. Discussion

Radiotherapy has long been a powerful strategy against cancer cell proliferation; however, the malignant cancer cells often find ways to bypass cell death [7,29,30,31]. The utility of combinatorial treatment modalities, specifically radiosensitizers in cancer therapy, was first investigated in the early 1970s [32]. Recently, nanotechnology has paved the way towards the development of an innovative cancer therapy platform [33,34]. Owing to the possibility of modification of nanoparticle composition and surface coating, the functions and specificity of nanoparticles could be more flexibly customized [35,36,37].

In our previous study, we developed nanoparticles of HA and polyethylene glycol-gelatin (PEG-gelatin) to specifically target CD44^+^ PCa cells [28]. Previous studies have also shown that prostate cancer cells express elevated levels of CD44 [38,39], and CD44 has shown promise as a target for cancer cell therapy [40]. In addition, CD44-expressing cells exhibit stem cell-like properties [41,42] and could be an ideal target for selectively killing CSC [43]. Mounting evidence has suggested the likelihood of CSC involvement in radioresistance [44,45,46], supporting the utility of a CSC-targeted radiosensitizer to improve the efficacy of radiotherapy.

Many bacterial toxins have been extensively studied for their promising clinical applications [47,48,49,50,51]. A previous study has shown that human gingival squamous carcinoma cell line Ca9-22 transfected with *Actinobacillus actinomycetemcomitans*–CdtB-expressing plasmid manifested G2 phase arrest and cell growth inhibition [52]. Additionally, in our previous work, chitosan/heparin nanoparticle-encapsulating CdtB was proven to be effective in causing G2/M phase cell cycle arrest in gastric cancer cells and finally induced cell death [53]. The potential of CDT as a radiosensitizer also has been tested in PCa models [54]. As described before, CDT exerts a similar effect as radiation via CdtB entry into the nucleus, but with the precondition of CdtA and CdtC binding to the cell surface. To minimize the size of the particles for better cellular uptake efficiency and tumor permeability, nanoparticles allowing intracellular delivery were designed, and only CdtB (without CdtA and CdtC) was purified and encapsulated into the nanoparticles. In addition, PEG–gelatin nanoparticles possess preferable advantages such as biocompatibility, biodegradability, and cost-effectiveness [55,56]. Gelatin is a denatured protein obtained from animal structural collagen [57], which can properly form a complete structure with CdtB; in addition, the PEG modification provides protection against enzymatic damage and the mononuclear phagocytosis system [58], which allows sustained circulation.

In this study, we attempted to analyze the potential of CDT as a radiosensitizer and further exploited progressive nanotechnology to develop a novel combinatorial treatment for PCa. To determine the binding activity of HA-CdtB-NPs, we visualized the localization of HA-CdtB-NPs using immunofluorescence and found that most HA-CdtB-NPs attached to the target cells as early as 1 h after treatment. Moreover, the amount of CdtB entry into the cytosol and subsequently the cell nuclei with the HA/Arg-CS delivery system was the highest at 6 h after treatment. Notably, the results also indicated that HA-CdtB-NPs have a better CdtB delivery efficiency than CDT holotoxin. Hence, CdtB has the potential to cause cancer cell death by directing DSB, which makes it an effective radiosensitizer.

The enhancement of radiosensitivity was further examined by the combined effect of HA-CdtB-NPs and IR. DSB formation and the phosphorylation of downstream proteins such as CHK2, PARP, and γ-H2AX confirmed the activation of the ATM-dependent DNA damage signaling pathway [59,60,61]. PCa cells treated with IR showed positive results, while HA-CdtB-NP/IR presented the most synergistic effect. Furthermore, after 24-h treatment with HA-CdtB-NPs, a large proportion of PCa cells were paused at the G2/M phase, which has been revealed to be the most vulnerable stage for radiation [62,63,64].

Although our results showed that HA-CdtB-NPs exert potent cytotoxic effects in radioresistant PCa cells, further investigations are required to validate their medical application. For instance, even though previous research showed that HA has an equivalent cancer cell cytotoxicity to anti-CD44 antibodies, CD44 does not serve as the only receptor for HA and it may not be a specific biomarker for PCa cells [43]. Therefore, further in vivo studies aimed at analysis of their biodistribution, circulation time, and antitumor activities are warranted.

## 5. Conclusions

**Figure 7:**
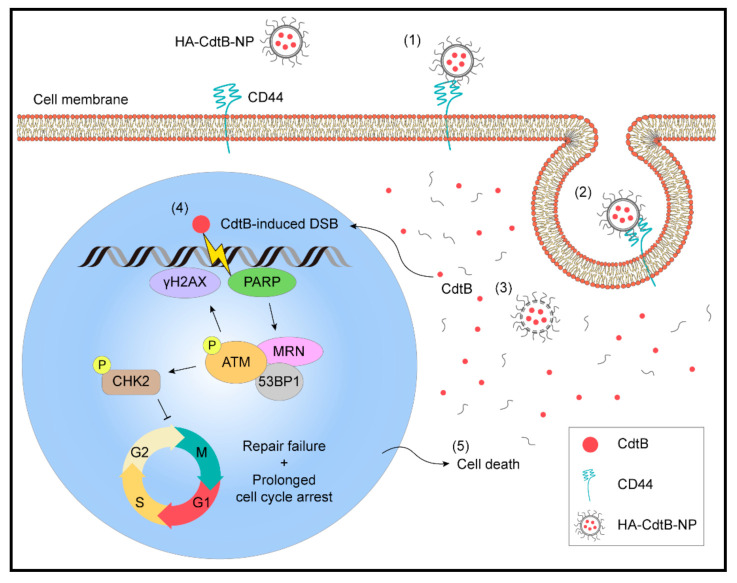
The effects of HA-CdtB-NPs on radioresistant PCa cells. (1) HA-based nanoparticles specifically bind to CD44 on the cell membrane and (2) the encapsulated CdtB is delivered into PCa cells through endocytosis. (3) With nuclear translocation signal (NLS), CdtB enters the nucleus and (4) exerts its DNase activity to effectively induce DSBs, leading to cell cycle arrest. (5) As the cancer cells fail to repair the extensive DNA damage and experience prolonged cell cycle arrest, they are sentenced to death. This study unveils the mechanism behind the radiosensitivity enhancement in PCa cells promoted by HA-CdtB-NPs and thus provides a promising target-specific agent for the development of a new therapy against radioresistant PCa.

In this study, we developed an HA-based nanoparticle delivery system to carry CdtB into PCa cells (Figure 7). Our results demonstrated that HA-CdtB-NPs possess activity similar to CDT holotoxin, but with superior advantages. The potential effects include maximum target specificity and delivery efficiency of CdtB into the nucleus and enhancement of the effect of IR in radioresistant PCa cells. Our results provide a novel strategy to employ HA nanoparticles encapsulated with CdtB as a radiation sensitizer, which can be developed as a potent therapeutic agent for radioresistant PCa.

## Figures and Tables

**Table 1:** Characteristics of prepared NPs constructed with differential HA:Arg-CS concentrations.

HA:Arg-CS (mg/mL)	Mean Particle Size (nm)	Polydispersity Indices	Zeta Potential Value (mV)
2.00:0.50	225.64 ± 14.65	0.23 ± 0.08	−38.79 ± 4.51
2.00:1.00	287.76 ± 37.78	0.35 ± 0.11	−27.72 ± 5.74
2.00:1.50	1165.98 ± 325.87	0.78 ± 0.21	−6.79 ± 3.24
2.00:2.00	298.28 ± 28.74	0.19 ± 0.12	+26.47 ± 2.95

**Table 2:** Characteristics of HA-NPs encapsulating varying CdtB concentrations.

CdtB:HA:Arg-CS (mg/mL)	Mean Particle Size (nm)	Polydispersity Indices	Zeta Potential Value (mV)	CdtB Loading Efficiency (%)
0.75:2.00:0.50	273.78 ± 17.37	0.27 ± 0.06	−38.89 ± 9.65	48.97 ± 5.12
0.50:2.00:0.50	261.43 ± 18.95	0.28 ± 0.08	−35.48 ± 7.32	45.79 ± 6.51
0.25:2.00:0.50	248.62 ± 20.16	0.21 ± 0.09	−35.13 ± 4.83	43.87 ± 4.98

## Data Availability

Please refer to suggested Data Availability Statements in section “MDPI Research Data Policies” at https://www.mdpi.com/ethics.

## Supplementary Materials

The following are available online at https://www.mdpi.com/2227-9059/9/2/151/s1, Figure S1: Fourier transform infrared analysis of Arg-CS, HA, and Arg-CS/HA nanoparticles, Figure S2: Analysis of CD44 expression in normal prostate cells (PZ-HPV-7) and radioresistant PCa cells (PC3-KD), Table S1: Loading efficiencies of HA-CdtB-NPs.
