# Supplementary material for: Bacterial Genotoxin-Coated Nanoparticles for Radiotherapy Sensitization in Prostate Cancer
Source: Biomedicines. 2021 Feb 4;9(2):151. doi: 10.3390/biomedicines9020151 (PMC7913852; doi:10.3390/biomedicines9020151)
Supplement: Supplementary file 1 [file biomedicines-09-00151-s001.pdf]

Bacterial Genotoxin-coated Nanoparticles for Radiotherapy Sensitization in Prostate Cancer

Supplementary material

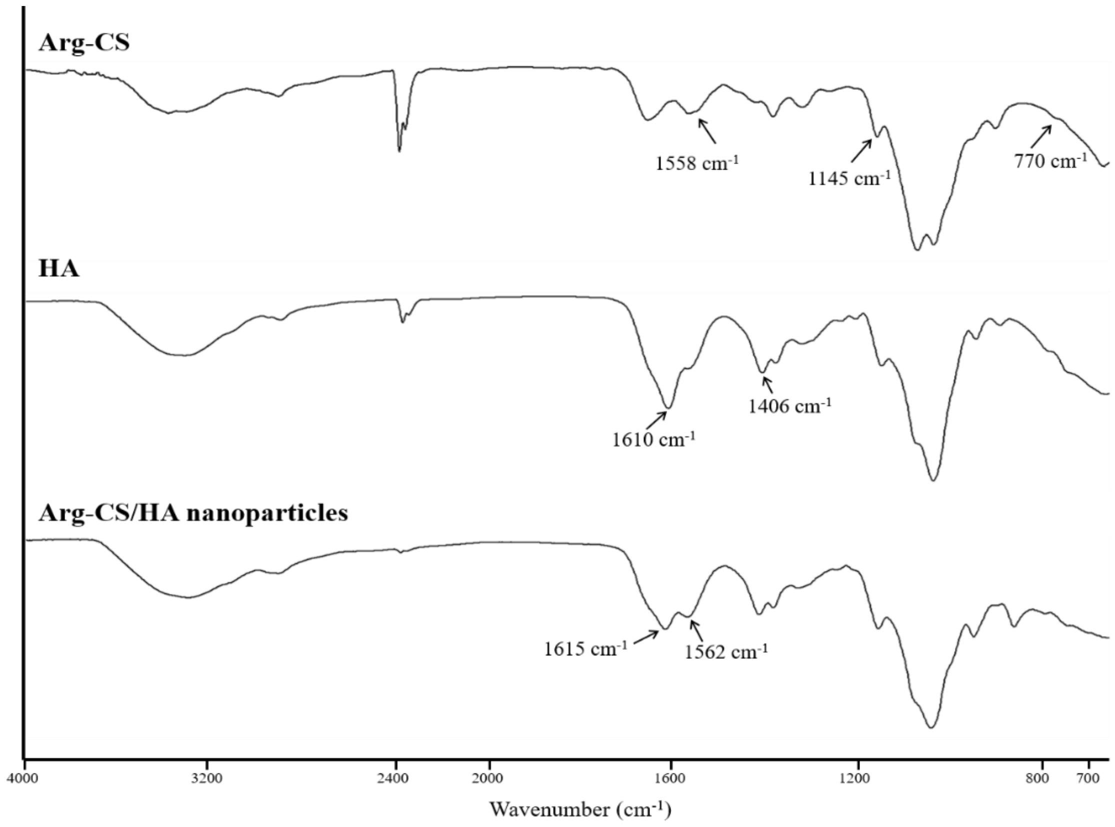

Figure S1. Fourier transform infrared analysis of Arg-CS, HA, and Arg-CS/HA nanoparticles.

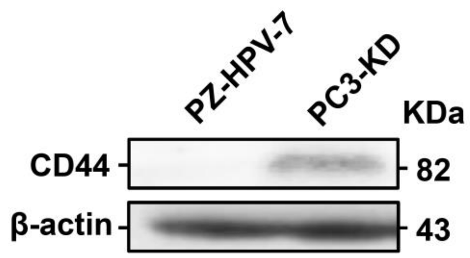

Figure S2. Analysis of CD44 expression in normal prostate cells (PZ-HPV-7) and radioresistant PCa cells (PC3-KD). CD44 expression levels in PZ-HPV-7 and PC3-KD cells were analyzed by western blot assay. β-actin was used as the loading control.

Table S1. Loading efficiencies of HA-CdtB-NPs.

Hyaluronic acid (μg/mL)	Loading efficiency (%)
0	76.95
312.5	68.08
625	60.34
1250	58.15
2500	72.48
5000	65.23
